# The diagnostic role and dynamic changes in cerebrospinal fluid neopterin during treatment of patients with primary central nervous system lymphoma

**DOI:** 10.1002/cam4.1581

**Published:** 2018-07-07

**Authors:** Mingying Geng, He Xiao, Jiaqi Liu, Yang Song, Ping Fu, Xing Cheng, Jinwei Zhang, Ge Wang

**Affiliations:** ^1^ Cancer Center Institute of Surgery Research Third Affiliated Hospital Army Medical University (Third Military Medical University) Chongqing China; ^2^ Department of Clinical Laboratory Institute of Surgery Research Third Affiliated Hospital Army Medical University (Third Military Medical University) Chongqing China; ^3^ Department of Pathology Institute of Surgery Research Third Affiliated Hospital Army Medical University (Third Military Medical University) Chongqin China; ^4^ Department of Neurosurgery Chongqing Cancer Hospital Chongqing China; ^5^ Department of Neurosurgery The Second Affiliated Hospital of Harbin Medical University Harbin Heilongjiang China

**Keywords:** biomarker, brain tumor, cerebrospinal fluid, inflammation, neopterin, primary central nervous system lymphoma

## Abstract

This study aimed at evaluating the diagnostic and prognostic role of neopterin (Npt) concentration in the cerebrospinal fluid (CSF) of patients with primary central nervous system lymphoma (PCNSL). Ninety‐nine patients were enrolled in this retrospective study; these included patients with PCNSL (n = 21), other brain tumors (n = 44), and inflammatory diseases (n = 34). CSF Npt concentration was measured using ELISA. Receiver operating characteristic (ROC) curve analysis was performed to assess the discriminative ability of CSF Npt concentration for the diagnosis of PCNSL. CSF Npt concentration in patients with PCNSL was significantly higher than that in patients with other brain tumors and inflammatory diseases (*P* < .001). On ROC curve analysis, the optimal cutoff CSF Npt level of 10.77 ng/mL for the diagnosis of PCNSL and the diagnostic yield of MRI were increased when used in conjunction with CSF Npt concentration. The CSF Npt concentrations in PCNSL patients with multiple lesions were significantly higher than those in patients with a single lesion. Changes in CSF Npt concentration were consistent with post‐treatment changes in tumor sizes. The CSF Npt concentration may be a good biomarker for the diagnosis, for monitoring of disease course, and for prognostic evaluation of patients with PCNSL.

## 1 INTRODUCTION

Primary central nervous system lymphoma (PCNSL) is an extranodal non‐Hodgkin's B cell lymphoma that originates in the brain tissue, leptomeninges, spinal cord, or eyes. The disease is typically confined to the central nervous system (CNS) with no extracranial extension.^1^ PCNSL is a rare tumor and accounts for approximately 3%‐5% of primary brain tumors. Approximately 90% of PCNSLs are diffuse large B‐cell lymphoma (DLBCL). During the past 25 years, the incidence of PCNSL has increased by approximately 3‐fold, especially in the HIV‐positive population.^2^ Although the pathogenesis of PCNSL remains unclear, it is believed that a systemically originating malignant clone of B cells is activated and homes into the immunologically privileged CNS, where the tumor cells undergo secondary mutations and proliferate.^3^ The PCNSL lesions most commonly involve the brain parenchyma. In a study, approximately 30%‐35% of patients with PCNSL were found to have multifocal lesions in the white matter, corpus callosum, basal ganglia, thalamus, and cerebellum.^4^ PCNSL often transiently responds to glucocorticoids, and the initial response to glucocorticoids may be used as a prognostic marker for PCNSL.^5^ PCNSL is commonly associated with a poor prognosis. High‐dose methotrexate‐based chemotherapy (H‐MTX) has greatly improved the prognosis of PCNSL patients as compared to that with whole‐brain radiotherapy (WBRT).^5^, ^6^ High‐dose chemotherapy followed by autologous stem cell transplantation as the first‐line treatment has been shown to improve 2‐year survival in elderly patients with PCNSL.^7^

Because patients with PCNSL commonly present with various nonspecific neurological symptoms, the diagnosis of PCNSL is sometimes difficult. Magnetic resonance imaging (MRI), especially contrast‐enhanced MRI, is the method of choice for the detection of PCNSL. However, as PCNSL does not have a characteristic radiological feature, misdiagnosis is not uncommon,^6^, ^8^, ^9^ and the differential diagnosis of PCNSL from other brain tumors such as meningioma, glioma, and metastatic brain tumors and some inflammatory diseases such as sarcoidosis and multiple sclerosis is typically challenging.^8^ Indeed, correct diagnosis of an atypical focal brain lesion based solely on radiological examination is a formidable challenge. Brain biopsy is the main diagnostic approach for PCNSL. However, brain biopsy is inadvisable for small or deeply located tumors and in patients at a high risk of hemorrhage or severe complications. In addition, diagnosis based on brain biopsy specimens obtained after craniotomy or stereotactic brain biopsy can result in a failure rate of approximately 10%‐35% in patients with rapidly deteriorating neurological conditions.^10^ Cytology or tumor biomarkers in the cerebrospinal fluid (CSF) are important for diagnosis and differential diagnosis of PCNSL, especially for patients who are not suitable for brain biopsy.^6^, ^11^ Identification of diagnostic biomarkers may help in the early diagnosis and treatment of PCNSL and improve patient prognosis.

Neopterin (Npt; 2‐amino‐6‐(1,2,3‐trihydroxypropyl)‐4(3H)‐pteridinone) is an intermediate metabolite in the process of biosynthesis of guanosine triphosphate (GTP).^12^ It is mainly synthesized and released by monocytes‐macrophages in response to the stimulation by cytokine interferon‐γ. It is stable in the body fluids such as blood, urine, CSF, and pleural fluid. Although the biologic characteristics of Npt remain unclear,^13^ Npt is regarded as a good biomarker of immune activation mediated by the “lymphocyte‐macrophage axis”.^14^ It is closely associated with cell‐mediated immune response in infectious states,^15^, ^16^ sepsis,^17^, ^18^ malignant tumors,^19^, ^20^, ^21^ and severe traumatic brain injury.^22^ However, it remains unclear whether Npt levels in the CSF can assist in the diagnosis of PCNSL. In this study, we compared the CSF Npt levels in combination with MRI and histological findings in patients with PCNSL, other brain tumors, and CNS inflammatory diseases. The purpose of this study was to investigate the diagnostic role of CSF Npt concentration in patients with PCNSL and to assess its use to distinguish PCNSL from other brain tumors and inflammatory diseases of the brain.

## 2 MATERIALS AND METHODS

### 2.1 Patients

This study was approved by the medical ethics committee at the local hospital. Written informed consent was obtained from all patients prior to their enrollment. This retrospective study included 99 patients with suspected PCNSL or inflammatory diseases of the CNS, who were hospitalized between October 2013 and October 2016 at the following hospitals: Departments of Neurosurgery, Neurology, Oncology, and Hematology at the Daping Hospital of the Third Military Medical University; Department of Neurosurgery at Chongqing Cancer Hospital; Department of Neurosurgery at the Second Affiliated Hospital of Chongqing Medical University; and Department of Neurosurgery at the Second Affiliated Hospital of Harbin Medical University. The inclusion criteria were as follows: (1) patients with suspected primary PCNSL, CNS inflammatory disease, or suspected metastatic encephaloma from other cancers for whom preoperative MRI or computerized tomography (CT) images were available; (2) HIV‐negative status; (3) CSF samples available; and (4) informed consent obtained from patients or their relatives (in the case of patients <18 years of age or those with mental disorders). The exclusion criteria were as follows : (1) age < 12 years; (2) history of trauma, malignant tumor, autoimmune diseases, or organ transplantation; (3) patients with symptoms of increased intracranial pressure, such as headache, nausea, vomiting, and optic disk edema owing to the increased risk of lumbar puncture; and (4) brain tissue deformation due to compression by tumor or peritumoral edema to prevent brain herniation; and (5) CNS metastasis from lymphoma identified by chest, abdomen, and pelvic examination.

### 2.2 Measurement of the CSF Npt concentration

Cerebrospinal fluid (4‐5 mL) samples were obtained via lumbar puncture for routine CSF examination prior to the start of any treatment. Only 2 patients (case 8 and case 20) had received brief steroid therapy before enrollment. The typical interval between CSF collection and MRI examination was <1 week. Two hours after CSF collection, CSF samples were centrifuged at 2000 *g* for 10 minutes, and the supernatants were frozen and stored at −80°C. The concentration of Npt in the CSF was measured using the human neopterin (Npt) ELISA kit (MyBioSource, LLC, CA, USA, Cat No. MBS2600934), according to the manufacturer's instructions. This Npt kit had a detection range of 0.312‐20 ng/mL, with a minimum detection limit of 0.06 ng/mL. The storage time of CNS samples ranged from 3 to 72 days.

### 2.3 MRI

All patients underwent MRI with a 3.0T Verio device (Siemens AG, Erlangen Germany). MRI scans were performed in 8‐channel head‐and‐neck coils. The MR sequences used in this study included T1‐weighted image (T1WI), T2‐weighted image (T2WI), fluid‐attenuated inversion recovery (FLAIR), diffusion‐weighted imaging (DWI), perfusion‐weighted imaging (PWI), and magnetic resonance spectroscopy (MRS). Enhanced MRI was performed after intravenous injection of gadolinium diethylenetriamine pentaacetic acid (Gd‐DTPA; 0.1 mmol/kg) at the rate of 3 mL/s. All data were transferred to Siemens workstation for further processing.

### 2.4 Evaluation

True‐positive PCNSL (L‐L) was defined by the diagnosis of PCNSL by both MRI and histology. False‐positive PCNSL (L‐NL) was defined by the diagnosis of PCNSL by MRI, but not by histology. False‐negative PCNSL (NL‐L) was defined by the diagnosis of PCNSL by histology, but not by MRI. True‐negative PCNSL (NL‐NL) was defined by the diagnosis of non‐PCNSL by both MRI and histology.

All patients underwent MRI and measurement of the CSF Npt concentrations before and after surgery, and during the follow‐up period of 1, 3, and 6 months. Complete remission (CR) was defined by post‐treatment disappearance of tumor mass on enhanced MRI. Partial remission (PR) was defined by post‐treatment decrease in tumor size (measured by the sum of the maximal diameter of the lesion) by ≥50% on enhanced MRI. Stable disease (SD) referred to a decrease in tumor size by <50% or an increase in tumor size by ≤25% on enhanced MRI after treatment. Progressive disease (PD) referred to an increase in tumor mass by >25% on enhanced MRI after treatment.

### 2.5 Statistical analysis

Statistical analyses were performed using SPSS 21.0 software (SPSS, Chicago, IL, USA). Quantitative data with normal distribution such as age and KPS are expressed as mean ± SD and the between‐group differences assessed with analysis of variance (ANOVA). Non‐normally distributed quantitative variables such as the CSF Npt concentration and biochemical data are expressed as median and range, and between‐group differences assessed by nonparametric Kruskal‐Wallis test, followed by post hoc Mann‐Whitney *U* test. Spearman's correlation analysis was performed to assess the relationship between Npt concentration and other biochemical CSF parameters. Receiver operating characteristic (ROC) curve analysis was performed to assess the discriminative ability of CSF Npt concentration for the diagnosis of PCNSL. Probability values <.05 were considered statistically significant.

## 3 RESULTS

### 3.1 Baseline characteristics

This study enrolled 99 patients including 65 patients with brain tumors and 34 patients with CNS inflammatory diseases. All brain tumors were surgically removed, and the diagnosis was confirmed by histopathological examination.

Of the 65 patients with brain tumors, 21 patients had PCNSL, including 20 patients with DLBCL and one patient with T‐cell lymphoma. Of the 21 patients with PCNSL, MR images showed multiple supratentorial lesions in 9 patients, while a single lesion was observed in 12 patients (1 orbital tumor, 2 infratentorial tumors, and 9 supratentorial tumors). Based on the MR images, the average number of tumors was 2 (range, 1‐7) and the mean total added tumor volume was 3.15 cm^3^ (range, 0.41‐21.3 cm^3^). Two patients received steroids before examination. Five patients were continuously followed up with complete data on MRI of the tumors and the CSF Npt concentrations.

Of the 44 patients with other brain tumors, 19 patients had newly diagnosed glioma; these included 4 patients with grade III glioblastoma, 6 patients with anaplastic glioma, 8 patients with diffuse astrocytoma, and 1 patient with pilocytic glioma. Meningioma was diagnosed in 9 patients, while 4 patients had metastatic brain tumors from lung cancer. The remaining 12 patients included 4 patients with schwannoma, and 1 patient each with olfactory neuroblastoma, embryoma, vascular malformation, pituitary tumor, craniopharyngioma, chordoma, medulloblastoma, and germ cell tumor. Five patients had received dexamethasone treatment prior to the collection of CSF specimens.

For the 34 patients with CNS inflammatory diseases, the specific diagnosis was viral encephalitis (n = 12), purulent meningitis (n = 2), allergic meningitis (n = 1), multiple neuritis (n = 1), unclassified encephalitis (n = 3), cryptococcal encephalitis (n = 4), multiple sclerosis (n = 3), encephalomalacia (n = 3), cyst (n = 2), fungal infection (n = 1), Creutzfeldt‐Jakob disease (n = 1), and cerebral amyloid angiopathy (n = 1).

### 3.2 Npt concentration in CSF is elevated in patients with PCNSL

**Figure 1:**
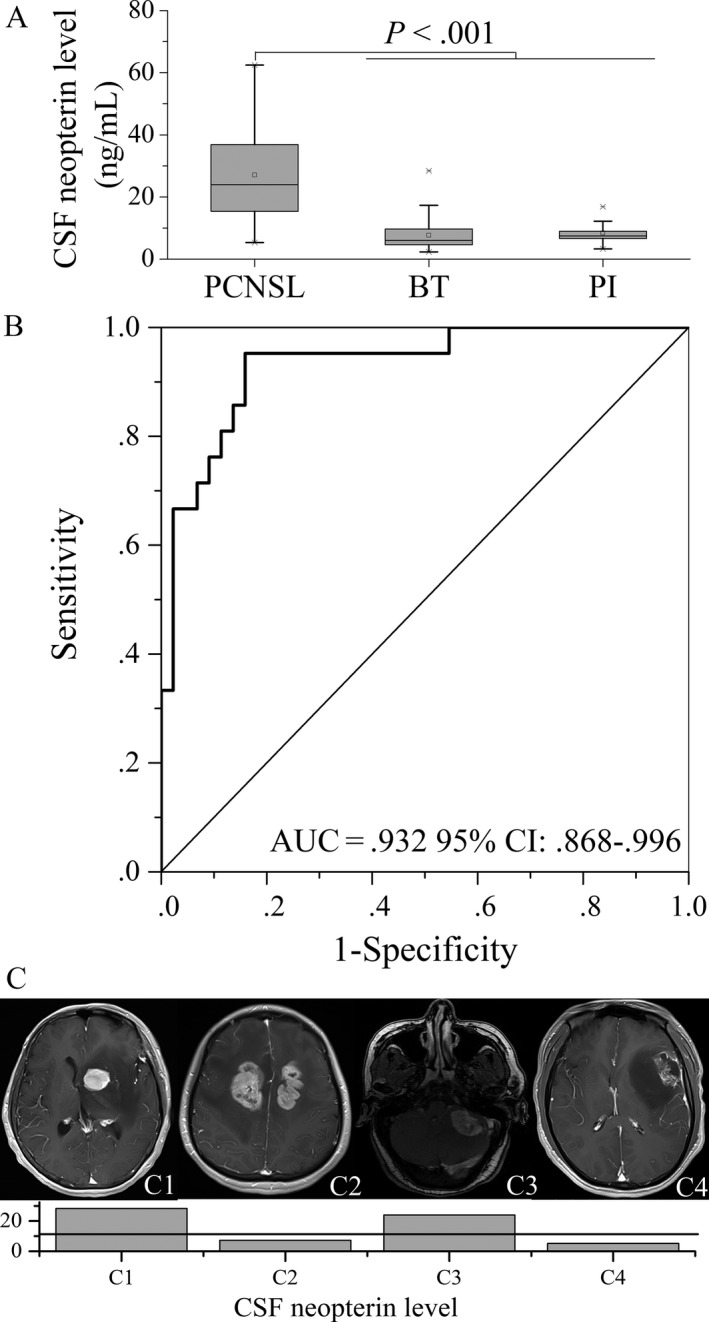
Diagnostic performance of CSF Npt concentration. A, Box plot of CSF neopterin concentrations in patients with PCNSL, other brain tumors (OBT), and inflammatory diseases (ID). Data are presented as median and range. *P* < .001 for PCNSL vs OBT and PCNSL vs ID. B, Receiver operating characteristic (ROC) curve was used to determine the cutoff value of neopterin level in the CSF to distinguish PSNCL from other brain tumor types in 65 patients with brain tumors. The area under the curve (AUC) was determined. C, Representative MR images and Npt concentrations in CSF in a true‐positive PCNSL case (C1), a false‐positive PCNSL case (C2), a false‐negative PCNSL case (C3), and a true‐negative PCNSL case (C4). C1 Npt: 28.291 ng/mL; C2 Npt: 7.229 ng/mL; C3 Npt: 23.976 ng/mL; and C4 Npt: 5.118 ng/mL

Table 1 summarizes the age, sex, Karnofsky Performance Status score (KPS score), and CSF biochemical parameters including total cell count, white blood cell (WBC) count, lactate dehydrogenase (LDH), glucose, protein, and the CSF Npt concentrations in all patients. There were significant differences between the 3 groups with respect to age, KPS score, total cell count, WBC count, LDH, glucose, and CSF Npt concentration (Table 1). CSF Npt concentration in patients with PCNSL was significantly higher than that in patients with other brain tumors (23.98 and 6.05 ng/mL, respectively; *P* < .001) and in patients with inflammatory diseases (7.50 ng/mL, *P* < .001; Figure 1A). CSF Npt concentration showed a negative correlation with total cell counts (*r* = −.199, *P* = .048) and WBC counts (*r* = −.245, *P* = .014) in all patients. No significant correlation between the CSF Npt concentration and other parameters was found.

**Table 1:** Demographic and CSF data in patients with PCNSL, other brain tumors, and inflammatory diseases

	PCNSL (n = 21)	Other brain tumors (n = 44)	Inflammatory diseases (n = 34)	*P*
Sex				
Female	9	21	18	.761
Male	12	23	16	
Age, median (range)	54 (36‐75)	47 (19‐81)	45.5 (18‐76)	.019
KPS score, median (range)	70 (50‐90)	85 (60‐90)	75 (30‐90)	<.001
Cell count, median (range) × 10^9^/L	0.012 (0.000‐1.104)	0.191 (0.001‐47.995)	0.114 (0.002‐52.640)	.006
WBC count, median (range) × 10^9^/L	0.006 (0.000‐0.032)	0.022 (0.000‐21.400)	0.016 (0.001‐4.160)	.001
LDH, median (range) U/L	34.2 (14.2‐319.9)	53.2 (8.1‐393.1)	23.6 (7.7‐1594.8)	.037
Glucose, median (range) mmol/L	3.34 (1.84‐7.69)	2.66 (0.41‐4.67)	3.29 (0.08‐4.45)	.018
Protein, median (range) g/L	0.91 (0.24‐11.29)	0.73 (0.16‐3.09)	0.375 (0.12‐19.00)	.120
Neopterin, median (range) ng/mL	23.98 (5.32‐62.47)	6.05 (2.32‐28.43)	7.50 (3.30‐16.87)	<.001

PCNSL, primary central nervous system lymphoma; KPS, Karnofsky Performance Status; WBC, white blood cell; LDH, lactate dehydrogenase.

### 3.3 Diagnostic performance of CSF Npt concentration

ROC curve analysis was used to evaluate the role of the CSF Npt concentration in discriminating PCNSL from other brain tumors. The results showed that the CSF Npt concentration was able to discriminate PCNSL from other brain tumors in 65 patients with brain tumors (Figure 1B). The optimal cutoff value of 10.77 ng/mL for the diagnosis of PCNSL was associated with 95.2% sensitivity, 84.1% specificity, 74.1% positive predictive value, and 97.4% negative predictive value (AUC = 0.932, 95% CI: 0.868‐0.996). The cutoff value of 10.77 ng/mL was able to discriminate PCNSL from other brain tumors and inflammatory diseases in all 99 patients with 95.2% sensitivity, 83.3% specificity, 60.6% positive predictive value, and 98.5% negative predictive value.

### 3.4 Association of CSF Npt concentration with radiological findings and tumor number

We further examined the association of the CSF Npt concentration with MRI findings for the diagnosis of PCNSL. Of the 21 histologically confirmed cases of PCNSL, correct diagnosis of PCNSL by MRI was made only in 11 patients. The sensitivity of MRI for the diagnosis of PCNSL was 52.4% (11/21). Of the 10 false‐negative PCNSL cases, 9 patients had CSF Npt concentrations greater than the cutoff value (10.77 ng/mL). The sensitivity of CSF Npt concentration for the diagnosis of PCNSL was 90% (9/10). Of the 44 patients with other brain tumors, correct diagnosis of non‐PCNSL by MRI was made in 38 cases. The sensitivity of MRI for the diagnosis of non‐PCNSL was 86.4% (38/44). Of the 6 false‐positive PCNSL cases, only one patient had CSF Npt concentration greater than the cutoff value. The sensitivity of CSF Npt concentration for the diagnosis of non‐PCNSL was 83.3% (5/6). Of the 38 true‐negative PCNSL cases, 6 patients had CSF Npt concentrations greater than the cutoff value. The sensitivity of CSF Npt concentration for the diagnosis of non‐PCNSL was 84.2% (32/38). Figure 1C shows the representative MRI mages and the CSF Npt concentrations of a true‐positive PCNSL case, a false‐positive PCNSL case, a false‐negative PCNSL case, and a true‐negative PCNSL case. CSF Npt concentration of >10.77 ng/mL increased the diagnostic yield of MRI for PCNSL, but did not increase its specificity.

Cerebrospinal fluid Npt concentrations in patients with multiple PCNSL lesions were significantly higher than those in patients with a single PCNSL lesion (median: 28.21 and 20.48 ng/mL, respectively, *P *=* *.035). Of the 21 patients with PCNSL, 20 (95.2%) patients had CSF Npt concentration greater than the cutoff value of 10.77 ng/mL. Only 1 patient (case 4 in Table 2), who received steroids before examination, had CSF Npt concentration <10.77 ng/mL (5.317 ng/mL), and the CSF sample was thawed twice for more than 12 hours.

**Table 2:** Baseline characteristics of 21 patients with PCNSL

Case	Sex/Age	Region	Single/multiple	Preoperative MRI diagnosis	Pathology	KI‐67 (%)	Npt (ng/mL)	LDH (U/L)	Treatment	Response	Survival
1	M/44	Left frontal	Single	Glioma; lymphoma	DLBCL	80	21.743	256.5	MTX/WBRT	CR	Yes
2	M/68	Right temporal/parietal	Single	Lymphoma	DLBCL	70	46.739	146.7	WBRT	CR	No
3	M/67	Left frontal/parietal/temporal	Multiple	Multiple metastatic tumors	DLBCL	50	11.012	141.5	MTX	PR	No
4	F/54	Sellar	Single	Pituitary adenoma	TCL	40	5.317	175.2	GKS/TMZ	CR	Yes
5	F/45	Right frontal	Single	Lymphoma	DLBCL	60	22.085	187.3	MTX/WBRT	CR	Yes
6	M/78	Right frontal/parietal	Multiple	Multiple meningioma	DLBCL	60	38.446	201.2	GKS/TMZ	PR	No
7	M/49	Left parieto‐occipital	Single	Lymphoma; angiogenic tumor	DLBCL	60	11.084	186.2	WBRT/TMZ	CR	Yes
8	F/61	Optic chiasm//left parietal	Multiple	Lymphoma	DLBCL	60	62.474	179.2	Steroid	PD	No
9	F/75	Right frontoparietal	Single	Lymphoma	DLBCL	80	12.594	214.2	WBRT	CR	Yes
10	M/52	Bilateral frontal	Single	High‐grade glioma	DLBCL	90	20.457	189	MTX/WBRT	CR	Yes
11	F/63	Left basal ganglion	Single	High‐grade glioma	DLBCL	40	28.291	183.8	GKS	CR	No
12	M/59	Corpus callosum/bilateral parietal	Multiple	Lymphoma	DLBCL	80	16.883	1501	MTX/WBRT	CR	Yes
13	M/67	Bilateral cerebral	Multiple	Metastatic tumors	DLBCL	70	43.746	176	MTX	CR	Yes
14	M/59	Bilateral cerebellar	Multiple	Bilateral cerebellar tumor	DLBCL	20	26.968	138.6	MTX/WBRT	CR	No
15	M/36	Right trigone of the ventricle	Multiple	Meningioma; neuroma; glioma	DLBCL	80	36.833	86.4	MTX/WBRT	CR	Yes
16	F/68	Right parietal	Single	Lymphoma; glioma	DLBCL	90	13.442	203.9	WBRT	CR	No
17	F/45	Right frontal/left occipital	Multiple	Lymphoma	DLBCL	50	13.358	187.7	MTX/WBRT	CR	Yes
18	M/46	Right frontal/parietal	Multiple	Lymphoma	DLBCL	40	55.754	190.1	Steroid	PD	No
19	F/49	Left frontal/right occipital	Multiple	Lymphoma; metastatic tumor	DLBCL	60	29.461	161.1	MTX/WBRT	CR	Yes
20	M/51	Right parietal	Multiple	Suspected inflammatory lesions	DLBCL	50	25.582	647.9	Steroid	PD	No
21	F/44	Bilateral basal ganglion/cerebellum	Multiple	Infection; vascular lesion; tumor	DLBCL	60	23.976	224.8	WBRT/TMZ	CR	Yes

M, male; F, female; MTX, methotrexate; WBRT, whole‐brain radiotherapy; GKS, Gamma Knife surgery; TMZ, temozolomide; CR, complete remission; PR, partial remission; PD, progress disease.

Of the 44 patients with non‐PCNSL tumors, 9 (20.45%) patients had CSF Npt concentrations greater than the cutoff value (10.77 ng/mL); these included 3 patients with glioblastoma, 3 patients with grade II astrocytoma, and 1 patient each with meningioma, olfactory neuroblastoma, and vascular malformation. No characteristic MRI findings were observed in these 9 patients.

Of the 34 patients with inflammatory diseases, 6 (17.6%) patients had CSF Npt concentrations greater than the cutoff value; these included 2 patients with purulent meningitis, 1 patient with allergic meningitis, 1 patient with multiple neuritis, and 2 patients with viral encephalitis.

### 3.5 Dynamic changes of CSF Npt concentration in patients with PCNSL

Three patients (Cases 2, 8, and 18) who had high CSF Npt concentrations (46.7, 62.5, and 55.754 ng/mL) died at day 3, day 27, and 8 months after surgery, respectively (Table 2).

**Figure 2:**
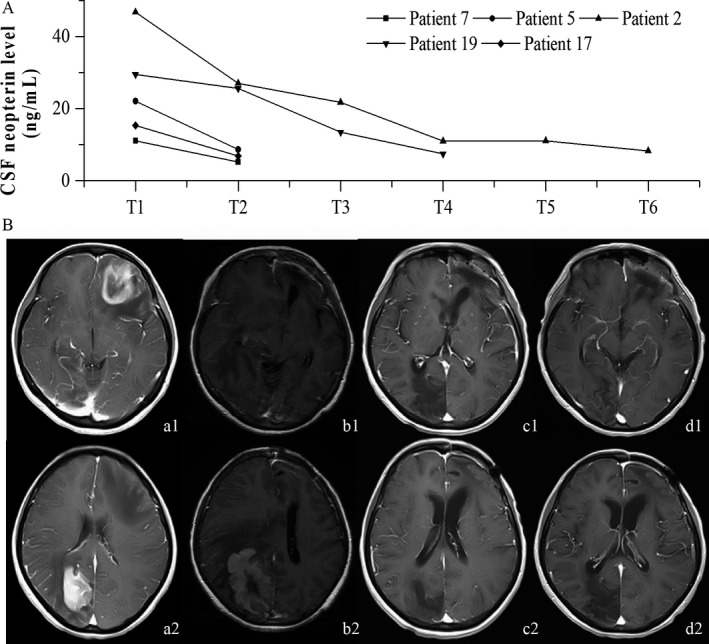
Monitoring tumor dynamics with CSF Npt concentration. A, Changes in the CSF Npt concentration before (T1) and immediately after (T2) surgery and at 1 (T3), 3 (T4), 6 (T5), and 9 (T6) months after surgery in 5 patients (Cases 2, 5, 7, 17, and 19). B, MR images and CSF Npt concentration in a 49‐y‐old woman with PCNSL

Five patients (Cases 2, 5, 7, 17, and 19) underwent regular MRI examination and assessment of CSF Npt concentration during the follow‐up. For these 5 patients, changes in CSF Npt concentrations were consistent with the changes in tumor size on MRI (Figure 2A). MRI showed CR in the 5 patients, and the CSF Npt concentrations were <10.77 ng/mL after treatment for 3 months. In Case 2, the Npt concentration decreased from preoperative 46.739 ng/mL to postoperative 26.968 ng/mL and further decreased to 11.012 ng/mL after whole‐brain radiotherapy (12 times, 24 Gy). Radiotherapy was stopped due to disease aggravation. At the 1‐month follow‐up, MRI showed PR and the CSF Npt concentration was 11.084 ng/mL. At the 3‐month follow‐up, MRI showed CR and the CSF Npt concentration was 8.279 ng/mL. At the 9‐month follow‐up, the disease aggravated with signs of tumor recurrence on MRI. The patient was hospitalized in another hospital, and the CSF Npt concentration was not measured due to the critical condition of the patient. In Case 9 who had 2 lesions (one each in the left frontal region and right occipital region), the CSF Npt concentration was not obviously reduced at 1 month after removal of the tumor in the left frontal region (preoperative: 29.46 ng/mL; postoperative: 25.58 ng/mL), and the MRI showed enlargement of the tumor in the right occipital region. The patient was administered 6 cycles of methotrexate (MTX) and whole‐brain radiotherapy (36 Gy). After radiotherapy for 3 months, the CSF Npt concentration was decreased to13.44 ng/mL and MRI showed CR with considerable edema. Six months after whole‐brain radiotherapy, the CSF Npt concentration was further decreased to 7.44 ng/mL and MRI showed CR with no obvious reduction in edema (Figure 2B).

## 4 DISCUSSION

In this study, we assessed the CSF Npt concentration in patients with PCNSL, other brain tumors, and CNS inflammatory diseases. We found that CSF Npt concentration in patients with PCNSL was significantly higher than that in those with other brain tumors and inflammatory diseases. On ROC curve analysis, a cutoff CSF Npt concentration of 10.77 ng/mL was found to effectively discriminate PCNSL from other brain tumors. CSF Npt concentrations >10.77 ng/mL were associated with increased diagnostic yield of MRI for PCNSL. In addition, changes in the CSF Npt concentration were consistent with the changes in tumor size on MRI during follow‐up. Therefore, CSF Npt concentration may serve as a useful biomarker for the diagnosis and monitoring of disease progression in patients with PCNSL.

Npt is mainly released from monocytes‐macrophages on stimulation by interferon‐γ.^13^ In the brain, microglial cells are the monocytes‐macrophages that have been found to be the main source of Npt in the CSF.^23^, ^24^, ^25^, ^26^ As PCNSL is commonly associated with increased microglial cell infiltration, increased Npt concentration in the CSF may be due to its increased secretion from microglial cells. In addition, human astrocytes were shown to disrupt the integrity of the blood‐brain barrier by a gap junction‐dependent mechanism,^27^ which leads to the release of Npt into the extracellular fluid and CSF.^28^, ^29^ Furthermore, PCNSL tumor cells can secrete monocyte chemoattractant proteins which may induce the secretion of Npt from monocytes‐macrophages.^30^ Several studies have also reported that B lymphocytes can secrete Npt.^31^, ^32^ In the present study, we found that the CSF Npt concentration in patients with PCNSL was significantly higher than that in patients with other brain tumors and inflammatory diseases. The higher Npt concentration in patients with PCNSL may be due to its increased secretion by tumor cells and/or the result of strong immune reaction induced by microglial infiltration.

In our study cohort of 99 patients, the cutoff CSF Npt concentration of 10.77 ng/mL was able to discriminate PCNSL from other brain tumors and inflammatory diseases with 95.2% sensitivity, 83.3% specificity, 60.6% positive predictive value, and 98.5% negative predictive value. Twenty‐one patients with PCNSL who had far low concentration of Npt included 20 patients with DLBCL and only one patient with T‐cell lymphoma. Therefore, it appears that Npt is only useful for the diagnosis of PCNSL associated with DLBCL histology. A decrease in the positive predictive value was observed after inclusion of patients with inflammatory diseases in the analysis. This may be because the 6 patients with inflammatory diseases had CSF Npt concentrations greater than the cutoff value of 10.77 ng/mL. As inflammation is known to trigger cell‐mediated immune response, increased release of Npt from monocytes‐macrophages via T lymphocytes may have led to the increase in the CSF Npt concentration in these patients.^17^, ^33^

Several CSF biomarkers such as antithrombin III,^34^ microRNAs,^35^ CXCL13 and IL‐10,^36^ and Npt^19^ have been shown to be highly specific for the diagnosis of PCNSL. In the study, we found that the CSF Npt concentration >10.77 ng/ml was able to discriminate PCNSL from other brain tumors with a sensitivity of 95.2% and a specificity of 84.1%. In addition, we found that the concomitant use of CSF Npt concentration greatly improved the diagnostic yield of cases of PCNSL with MRI. Npt is brain‐derived and not associated with blood‐brain barrier dysfunction.^29^ The CSF Npt concentration is greatly higher than the serum Npt concentration.^29^, ^37^, ^38^ Moreover, serum Npt concentration cannot be used in place of CSF Npt concentration for the diagnosis of PCNSL, as the serum Npt concentration is affected by many factors such as viral or bacterial infection and renal diseases.^19^ We determined serum Npt concentrations in patients with PCNSL, other brain tumors, and inflammatory diseases and found no significant differences among these patients (data not shown).

MRI does not have a high specificity for distinguishing PCNSL from glioma and metastatic brain tumors^39^, ^40^ and often results in misdiagnosis.^6^, ^8^, ^9^ In the present study, the sensitivity of MRI for the diagnosis of PCNSL was 52.4% (11/21), whereas the sensitivity of CSF Npt concentration for the diagnosis of PCNSL was 90% (9/10) in the 10 false‐negative PCNSL cases. In addition, the sensitivity of MRI for the diagnosis of non‐PCNSL was 86.4% (38/44 patients with brain tumors), whereas the sensitivity of CSF Npt concentration for the diagnosis of non‐PCNSL was 83.3% in the 6 false‐positive PCNSL cases and 84.2% in the 38 true‐negative PCNSL cases. Therefore, the CSF Npt concentration increased the positive detection rate of PCNSL by MRI, but did not increase the specificity of MRI for the diagnosis of PCNSL. Based on our findings, the use of CSF Npt concentration may improve the correct diagnosis of PCNSL and help avert the risk of obtaining brain biopsy specimens via stereotactic brain biopsy and craniotomy.

In this study, 2 patients (Cases 8 and 18) died on days 3 and 27 after surgery due to rapid disease progression. Both patients had CSF Npt levels greater than the cutoff value. Delayed diagnosis and treatment of PCNSL may result in rapid disease progression, and early treatment is essential to prevent disease progression and even death in these patients. As the therapeutic regimen is greatly different between PCNSL and other brain tumors, early correct diagnosis is very important for the selection of therapeutic regimen and improvement of prognosis of patients with PCNSL. For patients with high preoperative CSF Npt concentration, early brain biopsy should be performed to confirm the diagnosis, which may help avoid craniotomy. Once the diagnosis of PCNSL is confirmed, high‐dose methotrexate should be promptly administered as the first‐choice treatment. In addition, methotrexate in combination with other chemotherapeutic agents,^41^ autologous stem cell transplantation,^42^, ^43^ or rituximab^44^, ^45^ have been found to improve prognosis of patients with PCNSL.

We also found that CSF Npt concentration in PCNSL patients with multiple lesions was significantly higher than that in patients with single lesion. The 3 patients (Cases 2, 8, and 18) who died at 3 days, 27 days, and 8 months after surgery had multiple lesions with a high CSF Npt concentration. These findings suggest that CSF Npt concentration increases with the increase in the number of tumors and may be associated with poor prognosis in PCNSL patients. It has been reported that a high serum Npt concentration is associated with poor prognosis in patients with lung cancer.^21^ As Npt is stable in the CSF, dynamic measurement of changes in the CSF Npt concentration may be valuable for monitoring disease progression and for prognostic assessment of patients with PCNSL.

In summary, we found that the CSF Npt concentration was higher in patients with PCNSL than in patients with other brain tumors and inflammatory diseases. CSF Npt concentration may be a valuable biomarker for the diagnosis of PCNSL.^19^, ^34^, ^35^, ^36^ For suspected PCNSL patients with low CSF Npt concentration, craniotomy should be performed to remove the tumor as soon as possible. For suspected PCNSL patients with high CSF Npt concentration, brain biopsy should be performed as early as possible to confirm the diagnosis of PCNSL. If brain biopsy is contraindicated, the diagnosis of PCNSL should be first considered based on the high CSF Npt concentration, MRI findings, and the clinical symptoms; high‐dose methotrexate should be promptly administered in these patients as the first‐choice treatment. Owing to the retrospective study design and the relatively small sample size, our conclusions should be confirmed by a prospective study with a large sample size.

## CONFLICT OF INTEREST

The authors declare that they have no conflict of interest.
